# The Modulating Effect of p-Coumaric Acid on The Surface Charge Density of Human Glioblastoma Cell Membranes

**DOI:** 10.3390/ijms20215286

**Published:** 2019-10-24

**Authors:** Marcin Andrzej Kruszewski, Joanna Kotyńska, Magdalena Kusaczuk, Miroslav Gál, Monika Naumowicz

**Affiliations:** 1 Faculty of Chemistry, University of Bialystok, K. Ciolkowskiego 1K, 15-245 Bialystok, Poland; kaligula9@gmail.com (M.A.K.); joannak@uwb.edu.pl (J.K.); 2 Department of Pharmaceutical Biochemistry, Medical University of Bialystok, Mickiewicza 2A, 15-222 Bialystok, Poland; mkusaczuk@wp.pl; 3 Department of Inorganic Technology, Faculty of Chemical and Food Technology, Slovak University of Technology, Radlinského 9, 812 37 Bratislava, Slovakia; miroslav.gal@stuba.sk

**Keywords:** p-coumaric acid, surface charge density, microelectrophoretic mobility measurements, glioblastoma cells

## Abstract

p-Coumaric acid (p-CoA), a phenolic acid belonging to the hydroxycinnamic acids family, is a compound with tentative anticancer potential. Microelectrophoretic mobility measurements conducted at various pH values of electrolyte solution were applied to study p-CoA effects on electrical properties of human glioblastoma cell membranes. The obtained results demonstrated that after the p-CoA treatment, the surface charge density of cancer cells changed in alkaline pH solutions, while no noticeable changes were observed in cell membranes incubated with p-CoA compared to control at acidic pH solutions. A four-equilibrium model was used to describe the phenomena occurring on the cell membrane surface. The total surface concentrations of both acidic and basic functional groups and their association constants with solution ions were calculated and used to define theoretical curves of membrane surface charge density versus pH. The resulting theoretical curves and the experimental data were compared to verify the reliability and validity of the adopted model. The deviation of both kinds of data obtained at a higher pH may be caused by disregarding interactions between the functional groups of cancer cells. Processes occurring in the cell membranes after their incubation with p-CoA can lead to disorders of existing equilibria, which result in changes in values of the parameters describing these equilibria.

## 1. Introduction

Throughout history, plants have been used as remedies to treat different types of illnesses with satisfying results. Nowadays, over 60% of the anticancer drugs are of natural origin, making bioactive molecules increasingly interesting for drug companies, even as prototypes of final formulations for anticancer drugs [1].

It has been evident that plant extracts display anticancer activities in many cell lines. They usually exhibit more pronounced effects than pure natural or synthetic compounds. Despite remarkable efficiency of extracts, it is usually quite difficult to reliably determine which compound is interacting with the cancer cells or if the orchestrated effect is due to the synergistic interaction among the various compounds. Another problem associated with the use of extracts is the difficulty in obtaining enough quantity of plants to produce them, while the pure compounds can be synthesized in laboratory conditions [2].

Some of the biggest contributions in terms of pure molecules come from phenolic compounds, which are the most abundant secondary metabolites of plants which play important roles in both “in vitro” and “in vivo” studies [3]. They are widely distributed in the plant kingdom and, therefore, are an essential part of the human diet. Phenolic compounds are amphipathic molecules, containing at least one aromatic ring with one or more hydroxyl groups attached. Their molecular weight may range from low to very high and their structure may vary from simple to complex molecules. Within phenolic compounds, the following subgroups are distinguished: Xanthones, lignins, flavonoids, chalcones, lignans, coumarins, hydroxycinnamic acids, and hydroxybenzoic acids. All these families of compounds are recognized as having some sort of antitumor properties [3].

The hydroxycinnamic acids represent an important group of phenolic compounds derived from cinnamic acid through the phenilpropanoid pathway. The most abundant compounds within this group are ferulic, caffeic, and p-coumaric (p-CoA) acids. The hydroxycinnamic acids and their derivatives have shown broad biological and pharmacological activities [4,5,6]. In particular, the anticancer effect of caffeic and ferulic acids have been examined by several investigators [7,8,9]. Much less attention has been paid to p-CoA [10,11], which exists in a wide variety of edible plants such as tomatoes, navy beans, peanuts, carrots, barley, or garlic [12], and is also present in wine and vinegar [13]. Yet, a limited amount of data is currently available for p-CoA to comprehensively assess its bioavailability and in vivo effectiveness in humans [14].

It is already known that determination of the morphological and biochemical features of human cells is necessary to conduct fundamental research and perform a reliable assessment of the functioning of various organs and systems of the organism [15], or design new pharmaceuticals [16]. The literature indicates that the major part of electric properties in cells is dependent on cell membranes and cell composition [17]. Thus, it seems reasonable to believe that some structural alterations in cell membranes might occur under pathological conditions. Therefore, cancer cells are expected to have their own signature changes that are believed to affect their electrical properties. 

Electric properties of membranes are determined by acid-base equilibrium and sophisticated interactions between membrane components and surrounding elements. The equilibrium is set up by most of membrane molecules like phospholipids and proteins. At physiological pH, all mammalian cells have fixed negative charges on the surface, primarily due to carboxyl, phosphoric, and amino groups in their outer membranes [18]. These groups can easily be ionized as a function of environmental pH and contribute to the net charge of the cell surface. Determination of the surface charge of living cells is of prominent importance for understanding their behavior and functioning under various environmental conditions. Surface properties have provided information about cell surface composition, isoelectric point, rates of uptake of nutrients and drugs, as well as flocculation patterns of organisms. Membrane surface charges are carried by anionic phospholipids, however the exact nature of the lipids that empowers the cell membrane electrostatic field varies among eukaryotes [19]. Nevertheless, the surface charge density of membranes is not simply a function of the lipid composition, it also depends on environmental factors such as pH, temperature, and ionic strength [20].

The pH of the solution is a crucial factor in ensuring normal cellular function. Zhou and Raphael demonstrated that this parameter affects both mechanical and interfacial electrical properties of phosphatidylcholine membrane and that these alterations in membrane surface charge density and the Debye length can account for the experimentally measured changes in the membrane bending stiffness [21]. Pramanik et al. [22] studied the influence of ionic strength on the formation of supported vesicle layers of two anionic phospholipids 1,2-dimyristoyl-sn-glycero-3-phospho-rac-glycerol (DMPG) and dimyristoylphosphatidylserine (DMPS), onto gold. The authors found that at low ionic strength, low or no vesicle adsorption was observed as a result of vesicle–vesicle electrostatic repulsion. At medium ionic strength, the negative charges of DMPG and DMPS are screened, resulting in larger adsorption and a highly dissipative intact vesicle layer. In addition, DMPS exhibits a peculiar behavior at high ionic strength that depends on the temperature of the process [22]. 

The cell-surface charge is assessed by measuring zeta potential, which is the electrical potential of the interfacial region between the cellular surface and the aqueous region. Determination of zeta potential is particularly important since it is an indicator of the changes occurring on the surface of cell membrane. Zeta potential can be estimated by measuring cellular velocity or electrophoretic mobility in an electric field. 

In our previous article [23], the systematic studies investigating the effect of p-CoA on electrical properties of biological membrane models and the membranes of living cells were presented. Model membranes were made of three phospholipids asymmetrically distributed in the plasma membrane: Phosphatidylserine (PS)—the most abundant anionic phospholipid of the plasma membrane, which is tightly segregated to the internal leaflet of the plasma membrane in most cell types; phosphatidylethanolamine (PE)—neutral phospholipid also situated predominately in the internal leaflet; and phosphatidylcholine (PC)—neutral phospholipid, which appear to be mainly situated in the external leaflet. Moreover, the cytotoxic potential of p-CoA in human glioblastoma (GBM) cell lines was also evaluated. 

On the basis of previous experiments, here we utilized microelectrophoretic mobility measurements to extract a set of electrical-based parameters corresponding to the GBM cell status, treated versus non-treated with p-CoA, in both physiological and non-physiological conditions. Changes caused by p-CoA were monitored by determination of the electrical charge of the GBM membrane as a function of environmental pH, acid (*C*_TA_), and basic (*C*_TB_) functional group concentrations and their average association constants with hydrogen (*K*_AH_) or hydroxyl (*K*_BOH_) ions. Changes in values of these electrical parameters might be helpful in predicting overall influence of p-CoA on membranes of malignant cells, and hopefully, in the future, become a useful tool in determination of drug–membrane interactions while assessing therapeutic potential of particular agent. 

## 2. Theory

The pH dependence of the surface charge density of the cell membrane may adequately be characterized using a four-equilibrium model. Two equilibria concern the association of negative groups with the hydrogen and sodium ions, and two concern the association of positive groups with the hydroxide and chloride ions. The H^+^, OH^−^, Na^+^, and Cl^−^ ions are adsorbed on the cell membranes and the adsorption equilibria can be schematically described by the following equations [24]:

(1) $$A^{-}+H^{+}\Leftrightarrow \mathrm{AH}$$

(2) $$A^{-}+{\mathrm{Na}}^{+}\Leftrightarrow \mathrm{Ana}$$

(3) $$B^{+}+{\mathrm{OH}}^{-}\Leftrightarrow \mathrm{BOH}$$

(4) $$B^{+}+{\mathrm{Cl}}^{-}\Leftrightarrow \mathrm{BCl}$$

Thus, the association constants of the H^+^, OH^−^, Na^+^, and Cl^−^ ions with functional groups may be presented as:

(5) $$K_{\mathrm{AH}}=\frac{a_{\mathrm{AH}}}{a_{A^{-}}\cdot a_{H^{+}}}$$

(6) $$K_{\mathrm{ANa}}=\frac{a_{\mathrm{ANa}}}{a_{A^{-}}\cdot a_{{\mathrm{Na}}^{+}}}$$

(7) $$K_{\mathrm{BOH}}=\frac{a_{\mathrm{BOH}}}{a_{B^{+}}\cdot a_{{\mathrm{OH}}^{-}}}$$

(8) $$K_{\mathrm{BCl}}=\frac{a_{\mathrm{BCl}}}{a_{B^{+}}\cdot a_{{\mathrm{Cl}}^{-}}}$$

where *K*_AH_, *K*_ANa_, *K*_BOH_, *K*_BCl_ are association constants; *a*_A−_, *a*_AH_, *a*_ANa_, *a*_B+_, *a*_BOH_, *a*_BCl_ are surface concentrations of corresponding groups on the membrane surface; and *a*_H+_, *a*_Na+_, *a*_OH−_, *a*_Cl−_ are volume concentrations of solution ions.

The functional group concentration balances can be described as [24]:

(9) $$C_{\mathrm{TA}}=a_{A^{-}}+a_{\mathrm{AH}}+a_{\mathrm{ANa}}$$

(10) $$C_{\mathrm{TB}}=a_{B^{+}}+a_{\mathrm{BOH}}+a_{\mathrm{BCl}}$$

where *C*_TA_ is the total surface concentration of the membrane acidic groups and *C*_TB_ is the total surface concentration of the membrane basic groups.

The surface charge density is expressed by the equation presented by Dobrzyńska et al. [24]:

(11) $$\delta =\left(a_{B^{+}}-a_{a^{-}}\right)\cdot F$$

where *F* bears its usual meaning. 

When considering Equations (1)–(8) as a system of equations, it enables elimination of *a*_A−_, *a*_AH_, *a*_B+_, and *a*_BOH_ values from Equations (9)–(10) and *a*_A−_, *a*_B+_ from Equation (11). This allows one to obtain the equation describing surface charge of glioblastoma cell membranes [24]:

(12) $$\frac{\delta }{F}=\frac{C_{\mathrm{TB}}\cdot a_{H^{+}}}{\left(1+K_{\mathrm{BCl}}\cdot a_{{\mathrm{Cl}}^{-}}\right)+K_{\mathrm{BOH}}\cdot K_{W}}-\frac{C_{\mathrm{TA}}}{K_{\mathrm{AH}}\cdot a_{H^{+}}+K_{\mathrm{ANa}}\cdot a_{{\mathrm{Na}}^{+}}+1}.$$

Equation (12) must be simplified to a linear form at low H^+^ (*a*_H^+^_ → 0) and high H^+^ (*a*_H^+^_ → ∞) concentrations in order to determine the appropriate parameters. As a result of the simplification of the equation, two linear dependencies are obtained [24], one correct for low H^+^ ion concentration:

(13) $$\frac{\delta }{F}a_{H^{+}}^{-1}=\frac{-C_{\mathrm{TA}}\cdot a_{H^{+}}^{-1}}{1+K_{\mathrm{ANa}}\cdot a_{{\mathrm{Na}}^{+}}}+\left(\frac{C_{\mathrm{TB}}}{K_{\mathrm{BOH}}\cdot K_{W}}+\frac{K_{\mathrm{AH}}\cdot C_{\mathrm{TA}}}{{\left(1+K_{\mathrm{ANa}}\cdot a_{{\mathrm{Na}}^{+}}\right)}^{2}}\right),$$

and the other for high ion concentration:

(14) $$\frac{\delta }{F}a_{H^{+}}=\frac{C_{\mathrm{TB}}}{1+K_{\mathrm{BCl}}\cdot a_{{\mathrm{Cl}}^{-}}}\cdot a_{H^{+}}-\left(\frac{K_{\mathrm{BOH}}\cdot K_{W}\cdot C_{\mathrm{TB}}}{{\left(1+K_{\mathrm{BCl}}\cdot a_{{\mathrm{Cl}}^{-}}\right)}^{2}}+\frac{C_{\mathrm{TA}}}{K_{\mathrm{AH}}}\right).$$

In graphical representation, the intercepts and slopes can be easily determined from the above dependencies. The coefficients obtained from the linear regression may be applied to calculate *K*_AH_, *K*_BOH_, *C*_TA_, and *C*_TB_ values. Determination of values of these parameters enables subsequent calculation of the theoretical values of cell membrane surface from Equation (12) in comparison to experimental data. 

## 3. Results 

In order to get a deeper understanding of membrane/p-CoA interactions, the ability of p-CoA to alter the surface charge of four human glioblastoma cell lines was inspected by employing microelectrophoretic mobility measurements. The experiments were conducted at several pH using 0.9% NaCl, which served as a supporting electrolyte. Representative plots are presented based on at least three independent experiments.

The experimental data concerning surface charge of membranes of LN-18, LN-229, and LBC3 glioblastoma cell lines treated with p-CoA are available in [23]. Herein, we present the experimental values determined for one additional GBM cell line T98G. Moreover, the theoretical approach for analyzing the equilibria occurring in membranes of all four GBM cell lines incubated with p-CoA. as well as the determination of parameters characterizing these equilibria, are proposed. 

**Figure 1:**
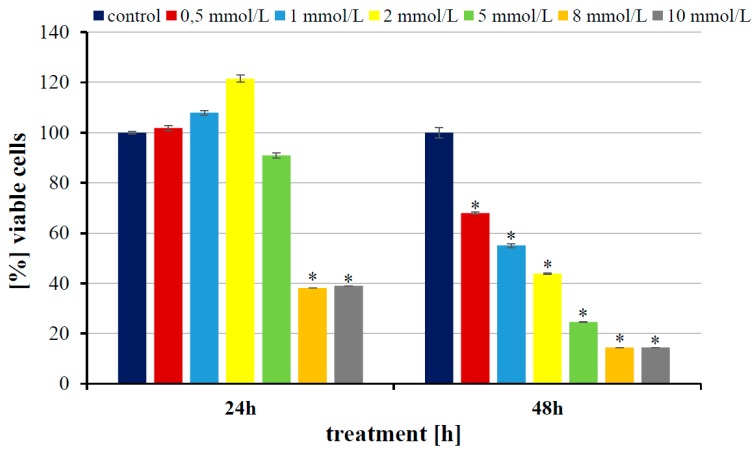
The viability of glioblastoma T98G cell line untreated and treated with 0.5, 1, 2, 5, 8, and 10 mmol/dm^3^ of p-coumaric acid for 24 and 48 h. The results represent mean values from three independent experiments ± SD. Significant changes are expressed relative to controls and marked with asterisks. Statistical significance was considered if * *p* < 0.05.

All tested cell lines were cultured with 5 and 8 mmol/dm^3^ of p-CoA for 24 and 48 h, based on the results of the MTT (3-(4,5-dimethylthiazol-2-yl)-2,5-diphenyltetrazolium bromide) tetrazolium reduction analysis. These concentrations correspond with high cytotoxic effect of p-CoA in all cell lines. The results of MTT analysis performed for T98G cells are presented in Figure 1, and cytotoxicity results for LN-18, LN-229, and LBC3 cells were described in our previous paper [23]. In all cases, untreated cells served as a control. 

**Figure 2:**
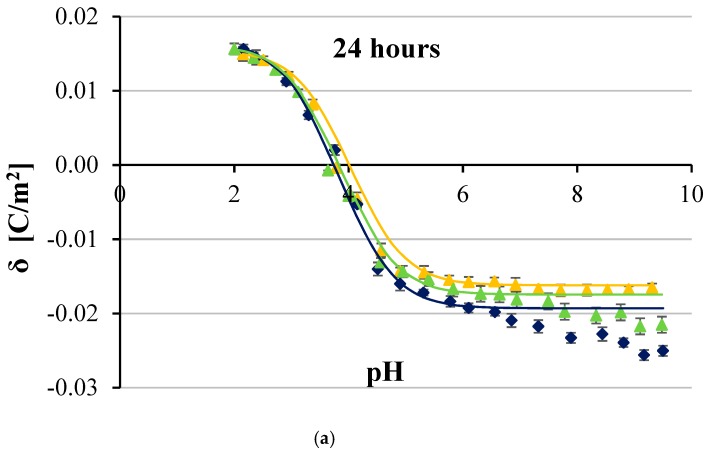

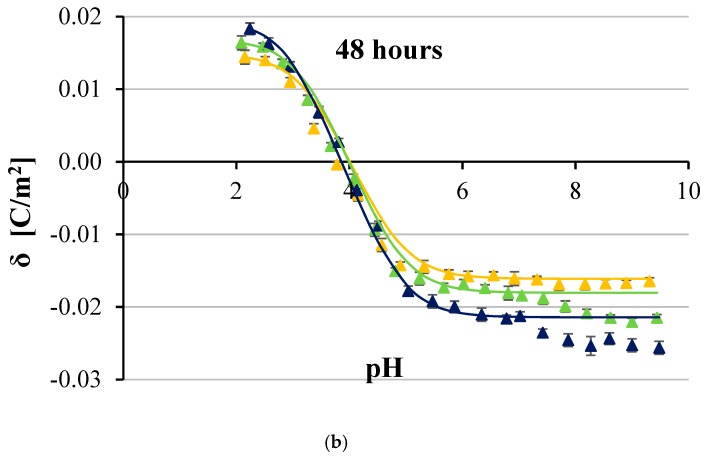
Dependence of surface charge density of glioblastoma LN-18 cell line as a function of the pH of the electrolyte solution. The cells were untreated (navy blue) or treated with 5 (green) and 8 (orange) mmol/dm^3^ of p-coumaric acid for 24 h (**a**) and 48 h (**b**). Solid lines describe theoretical values; experimental data are marked by points.

**Figure 3:**
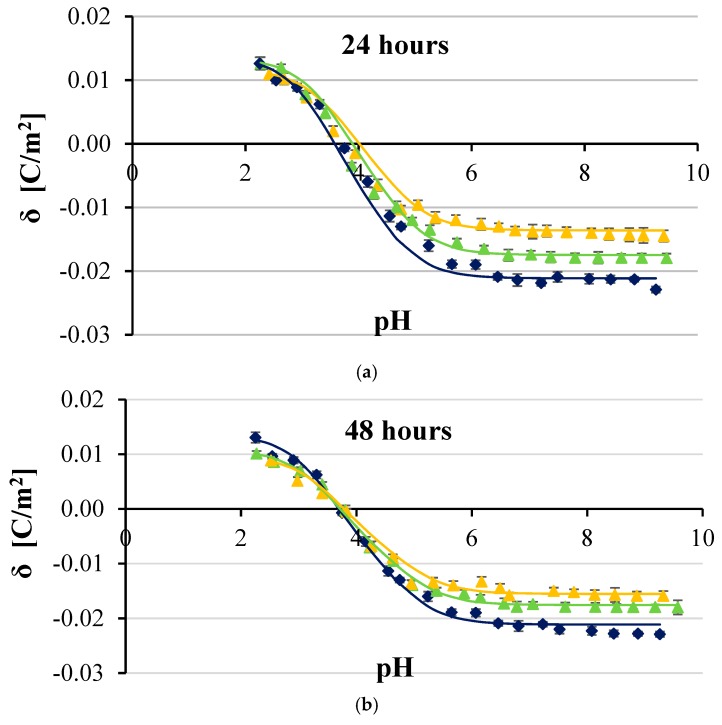
Dependence of surface charge density of glioblastoma LN-229 cell line as a function of the pH of the electrolyte solution. The cells were untreated (navy blue) or treated with 5 (green) and 8 (orange) mmol/dm^3^ of p-coumaric acid for 24 h (**a**) and 48 h (**b**). Solid lines describe theoretical values; experimental data are marked by points.

**Figure 4:**
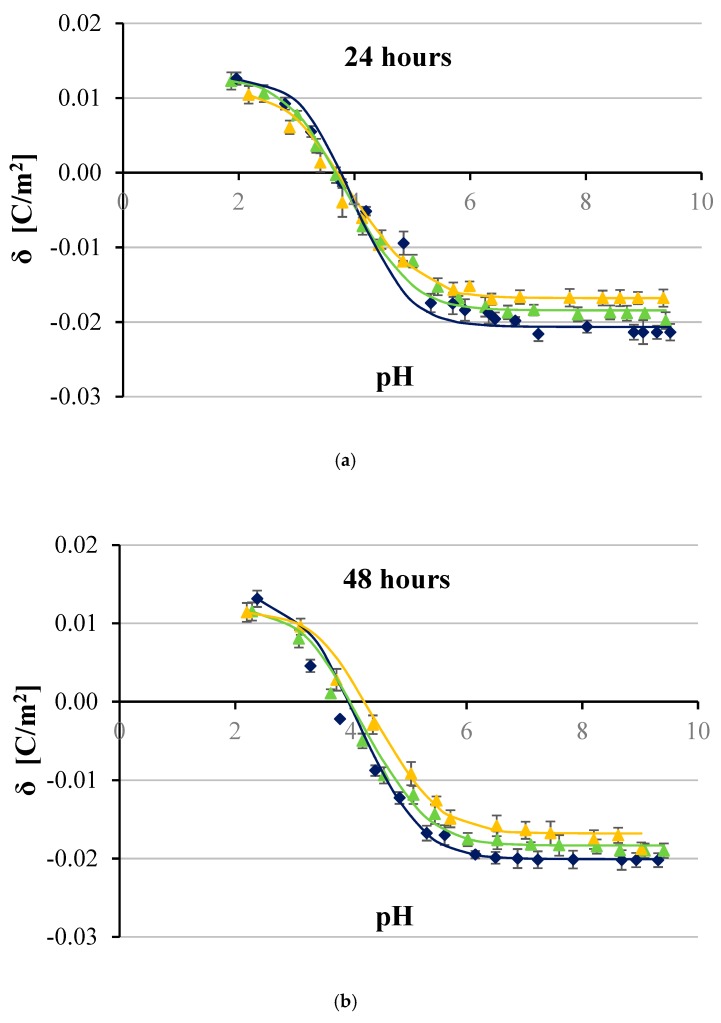
Dependence of surface charge density of glioblastoma LBC3 cell line as a function of the pH of the electrolyte solution. The cells were untreated (navy blue) or treated with 5 (green) and 8 (orange) mmol/dm^3^ of p-coumaric acid for 24 h (**a**) and 48 h (**b**). Solid lines describe theoretical values; experimental data are marked by points.

**Figure 5:**
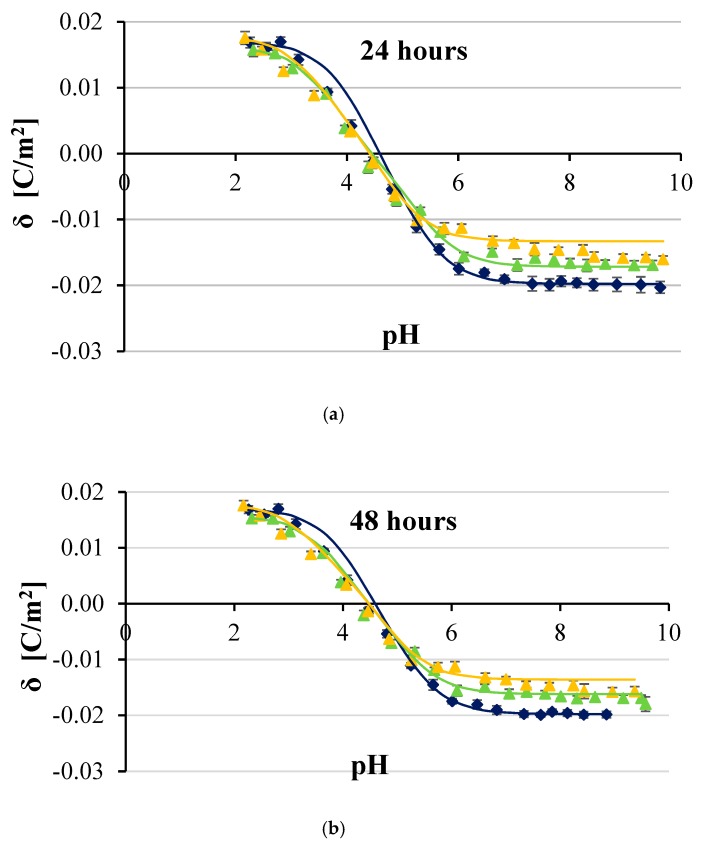
Dependence of surface charge density of glioblastoma T98G cell line as a function of the pH of the electrolyte solution. The cells were untreated (navy blue) or treated with 5 (green) and 8 (orange) mmol/dm^3^ of p-coumaric acid for 24 h (**a**) and 48 h (**b**). Solid lines describe theoretical values; experimental data are marked by points.

Figure 2, Figure 3, Figure 4 and Figure 5 show the effect of two concentrations of p-CoA (5 and 8 mmol/dm^3^) on the surface charge densities of LN-18 (Figure 2), LN-229 (Figure 3), LBC3 (Figure 4), and T98G (Figure 5) glioblastoma cell lines. The experimental values of the membrane surface charge densities were obtained from the electrophoretic mobility using Equation (15) and are expressed as points. The theoretical values of the membrane surface charge densities were calculated on the basis of Equation (12) and are plotted as curves in the figures.

It is apparent that similarly shaped curves characterizing the surface charge density dependencies of cancer cell membranes vs pH were obtained in all cases. Increasing acidity, corresponding with a decrease in pH values, results in an increasing positive surface charge density, but only to a certain value. However, increasing basicity associated with increasing pH causes the increase in negative surface charge density until the plateau is achieved.

Furthermore, it is obvious that at low pH values, no statistically significant changes were observed in the surface charge densities of membranes of any of the analyzed GBM cell lines treated with p-CoA compared with the control group. On the other hand, 24 h exposure to p-CoA of LN-18 (Figure 2a), LN-229 (Figure 3a), LBC3 (Figure 4a), and T98G (Figure 5a) cells results in increase in negative charge at high pH values compared with the control cells. Those alterations were apparently dose-dependent because greater amount of acid caused a greater increase in charge, which is reflected in the aforementioned figures. However, this trend was not evident in time—48 h exposure to CoA of LN-18 (Figure 2b), LN-229 (Figure 3b), LBC3 (Figure 4b), and T98G (Figure 5b) cells gave inconspicuous changes in the surface charge density values compared to 24 h exposure.

Finally, the results presented in Figure 2, Figure 3, Figure 4 and Figure 5 illustrate that the presence of p-CoA does not change the value of the isoelectric point of all analyzed GBM cell membranes tested at both 24 and 48 h exposures.

Besides experimental data, all figures present solid lines resulting from the previously characterized four-equilibrium model, which describes the adsorption of electrolyte ions on a cell membrane surface. In order to plot these lines, the values of physicochemical parameters characterizing GBM cell surfaces were obtained by adaptation of the model extensively explained in e.q. [25], and only briefly sketched herein. The total concentrations of functional acidic (*C*_TA_) and basic (*C*_TB_) groups in treated and untreated cancer cell membranes as well as their average association constants with hydrogen (*K*_AH_) and hydroxyl (*K*_BOH_) ions were determined by applying Equations (13) and (14). Determination of the above parameters was based on the assumption that the values of *K*_ANa_ and *K*_BCl_ association constants are the same as those obtained for phosphatidylcholine liposomes, which are 0.230 and 0.076 m^3/^mol, respectively [26]. Obtained *C*_TA_, *C*_TB_, *K*_AH_, and *K*_BOH_ values were incorporated into Equation (12) in order to calculate theoretical data of surface charge densities of analyzed GBM cell lines at various pH. The results, indicated in Figure 1, Figure 2, Figure 3 and Figure 4, seem to be calculated successfully as they show good alignment with the experimental data.

Table 1 lists the values of all the above-mentioned parameters calculated for LN-18, LN-229, LBC3, and T98G cell surfaces. The listed values derived from standard statistical analysis are reported as means ± standard deviation. It is clearly visible that treatment of these cells with p-CoA changes the physicochemical parameters characterizing their surfaces in comparison to the untreated cells. 

## 4. Discussion

Glioblastoma is the most common type of primary brain tumor in adults and the most lethal and least successfully treated tumor. The low absolute incidence combined with high morbidity, poor response rate, and short survival time poses practical problems for clinical trial execution [27]. Less than 30% of patients suffering from this devastating disease survive 12–15 months, even after receiving multimodal treatments such as surgical resection, combined chemotherapy and radiotherapy, and adjuvant chemotherapy [28]. These observations underscore the need for alternative therapies to prevent and effectively treat glioblastoma.

Epidemiological studies in humans have shown that regular consumption of fruits and vegetables is associated with reduced risk of cancer [29,30,31]. One possible explanation is the content of flavonoids, which exert anticarcinogenic activities [31,32]. p-Coumaric acid is a phenolic compound with tentative anticancer potential [33]. It has been shown to evoke its anticancer activity through reduction of proliferation, diminished adhesion, and suppressed migration of human cancer cell lines such as colon (HCT-15, HT29-D4), lung (A549), and glioblastoma [34,35,36].

As is well known, the mode of action of polyphenols is most likely dependent on their interaction with membranes. Thus, it seems reasonable to think that their functioning can be at least partially attributed to their specific effect on the membrane characteristics such as the ability to conduct ions, fluidity, and overall volume [37,38], as well as their specific interactions with individual lipid molecules and their arrangement inside the membrane [39]. The location of natural polyphenols in membranes remains controversial. Some experimental studies suggest that compounds with numerous polar OH groups are located deep inside membranes [38,40]. Other studies locate polyphenols closer to the membrane surface where they can interact with the polar head-groups of lipids through hydrogen bond formation [41,42]. The location of polyphenols inside membranes depends strongly on pH and polyphenols charge; the lower the pH, the lower the deprotonation state and the deeper the penetration [38]. It was evidenced that at pH equal to 7, the aromatic ring of the p-CoA is likely to be partially inserted into membrane [14].

Cellular membranes are characterized by a clearly ordered structure. Thus, they can be considered as a separate phase from a physical point of view—separated from surrounding cytoplasm or intermolecular biological fluid. Therefore, the membrane surface can be approximated to an interface. The membrane-surrounding solution interface is the place where physicochemical processes characteristic for a typical interface occur, e.g., asymmetric distribution of electric charge [43]. 

Examination of the electrical charge could reveal important information about the equilibria existing between membrane components as well as between them and their surroundings. Since there is an enormous number of these equilibria, it is necessary to adapt an appropriate number of parameters containing the mean values for all equilibria. The assumed parameters can be the total surface concentration of membrane acidic and basic groups on a membrane surface or the averaged association constants with solution ions. Determination of numerical values of these parameters is very important as it can help to better understand and describe changes in cellular membranes resulting from various factors and processes. 

In the present paper, both experimental and theoretical approaches for analyzing the effect of p-CoA on the electric charge density of human glioblastoma cell lines were applied. According to the results collected by using microelectrophoretic method, the change in surface charge was detected in all tested cell lines after p-CoA treatment compared to the intact cells in alkaline pH solutions, while there were no significant alterations at acidic pH solutions. Analysis of data presented in Figure 2, Figure 3, Figure 4 and Figure 5 reveals that theoretical and experimental results exhibit good agreement in the analyzed pH range. Occasional small differences at high pH values can be caused by the interactions occurring between the functional groups of the GBM membrane components, which were not considered in the proposed mathematical model. 

The determined values of the parameters describing the equilibria between membrane surface components and the surrounding ions (Table 1) indicate that p-CoA caused an increase of *C*_A_ and *C*_B_. The changes in functional group composition on the membrane surface are due to the appearance of new functional groups and/or to the disappearance of existing ones during the reaction of p-CoA with glioma membrane surface components. Variations in the number and kind of functional groups result in variations in *C*_A_ and *C*_B_, which in turn alter association constants of negatively charged (*K*_AH_) and positively charged (*K*_BOH_) groups.

According to the available data, specific interactions of polyphenols with negatively charged phospholipids rather than zwitterionic ones are more likely to appear in cell membranes [39]. This is in line with our previous results [23], where phosphatidylcholine, phosphatidylethanolamine, and phosphatidylserine liposomal surfaces were examined and the specific interactions of p-CoA with phosphatidylserine, which has a net of negative charge at physiological pH, were detected. Detection of these interactions is very valuable information, because phosphatidylserine exposition to the outer membrane surface, resulting in additional negatively charged groups, is the most pronounced effect of the lipid asymmetry disorders, the maintenance of which is extremely important for many cellular processes. The uncontrolled loss of the asymmetric distribution of phosphatidylserine in the membrane contributes to disturbances in cell functioning and is observed in many pathological changes. This process often occurs before the appearance of any other morphological symptoms associated with cell death. Studies of lipids conducted on melanoma and metastases have proven that the overall PS content of cancer cells did not increase in comparison to non-cancer counterparts, and only the asymmetric PS distribution in the plasma membrane was lost [44]. Likewise, Utsugi et al. [45] reported an elevated expression of PS in the outer leaflet of human tumor cells, whereas Ran et al. [46] demonstrated increased exposure of anionic phospholipids specifically on the surface of tumor blood vessels. These observations are additionally supported by the studies of Riedl et al. [44], who found that exposure of PS to the outer leaflet of membranes is a general phenomenon in cancer plasma membranes independent of cancer type.

To examine the mechanism of exposure of anionic phospholipids on tumor endothelial cells, a series of experiments was performed in which endothelial cells in vitro were treated with various factors and conditions known to be present in the tumor microenvironment [46]. It was concluded that anionic phospholipids on tumor vessels may be considered as target molecules for tumor therapy. 

The results presented in this paper, together with previous report demonstrating p-CoA-induced reduction in cell viability, ATP depletion, increase in caspases activity, and deregulation of gene transcription [23], suggest that p-CoA permeates and interacts with cellular and organelle membranes, which may be a key determinant of its effectiveness. These findings became a basis of more in-depth theoretical calculations describing the chemical nature of membrane-dependent electrical parameters. Acquired knowledge may imply that, in the future, changes in surface charge of membranes of living cells might not only serve as potential predictors of membrane permeability, but also indicate differential composition of membranes in various cells. As such, modern pharmacological analyses strive towards utilizing multidisciplinary approaches in designing and describing properties of potential drugs, which opens new opportunities for using chemical assessments in contemporary pharmacotherapy. Perhaps in-depth chemical studies will contribute to establishing novel approaches, allowing prediction of membrane permeability of the drug on the basis of computational analyses of electrochemical parameters. However, further studies are still required to evaluate the reliability of such analyses and develop universal mathematical equations matching various tested substances or being specific to particular type of cancer. In this respect, comprehensive studies performed for multiple components on various cancer cell lines are needed to outline the common pattern of such analyses to be useful tools of drug testing. Thus, although very speculative, our findings may encourage further examinations of electrochemical parameters of membranes in the context of pharmacological research. 

## 5. Materials and Methods

### 5.1. Materials and Reagents 

Human glioblastoma cell lines LN-18, LN-229, and T98G were supplied by the American Type Culture Collection (ATCC) (Manassas, Virginia, USA). LBC3 cell line was a kind gift of Prof. Cezary Marcinkiewicz (Department of Neuroscience, Temple University, Philadelphia, PA, USA), and was developed from *glioblastoma multiforme* tissue taken from a 56-year-old female patient subjected to surgical tumor resection [47]. p-Coumaric acid (Sigma, St. Louis, MO, USA, catalog no. 55823) was suspended in ethanol and further diluted with Dulbecco’s modified Eagle’s medium (DMEM) to the appropriate concentrations (0.5–10 mmol/dm^3^). Fetal bovine serum Gold (FBS Gold), DMEM, glucose, penicillin, and streptomycin for cell cultures were supplied by Gibco (San Diego, CA, USA), while ethanol was derived from POCH (Avantor Performance Materials, Gliwice, Poland). 

### 5.2. Cell Cultures and Treatment

All GBM cell lines were cultured as described earlier in our works [48,49]. Briefly, cell lines were grown in DMEM containing 10% fetal bovine serum, 4.5 mg/cm^3^ glucose, 100 μg/cm^3^ streptomycin, 100 U/cm^3^ penicillin, and 2 mmol/dm^3^ L-glutamine, and kept in an incubator (humidified atmosphere, 5% CO_2,_ 37 °C). Confluent cells were supplemented with p-CoA (0–10 mmol/dm^3^) and incubated for 24 and 48 h. Then, all cell lines were subjected to MTT analysis performed according to the method of [50], described in detail in our previous work [51]. Based on the MTT results, two concentrations of p-CoA (5 and 8 mmol/dm^3^) were chosen to proceed for further Z-potential examinations. 

### 5.3. Microelectrophoretic Mobility Measurements

Membrane mobility was assessed by performing microelectrophoretic measurements of samples using Zetasizer Nano ZS (Malvern Instruments, Worcestershire, UK) apparatus. The measurements were performed as a function of pH (in pH range 2–10), using 0.9% NaCl as a supporting electrolyte solution. The samples were suspended in alkali metal chloride solution and titrated to the desired pH with HCl or NaOH. The experiments were repeated at least three times, and data are presented as the mean ± standard deviation (SD).

The surface charge density *δ* was determined converting electrophoretic mobility values according to equation [52]:

(15) $$\delta \;=\;\frac{\eta \cdot u}{d}$$

where *η* is the viscosity of solution, *u is* the electrophoretic mobility, and *d* is the diffuse layer thickness.

The diffuse layer thickness was determined using the expression [53]:

(16) $$d\;=\;\sqrt{\frac{\varepsilon \cdot \varepsilon_{0}\cdot R\cdot T}{2\cdot F^{2}\cdot I}}$$

where *R* is the gas constant, *T* is the temperature, *F* is the Faraday constant, *I* is the ionic strength of 0.9% NaCl, and *ε* and *ε*_0_ refer to the permeability of the electric medium. 

## 6. Conclusions

In the present paper, we described the effects of p-CoA on electrical properties of membranes of four human glioblastoma cell lines (LN-18, LN-229, LBC3, T98G). We employed a theoretical approach to understand the equilibria occurring between membranes of cells incubated with p-CoA and their surroundings. The theoretical estimates of electric charge enabled the determination of the total concentrations of functional acidic and basic groups on treated and untreated cancer cell membranes as well as their average association constants with hydrogen and hydroxyl ions. As it can be seen, values of all the parameters determined for untreated cells are different in comparison to the cells incubated with p-CoA. The same dependencies were observed for each of the two concentrations of p-CoA (5 and 8 mmol/dm^3^) and for cells incubated for both 24 and 48 h. 

The course of strictly controlled processes in human organisms alters substantially under the influence of factors such as pharmaceuticals or alcohol and also in pathological conditions of the body including cancers and cardiovascular diseases. Any changes in the functioning of the cell translate into the functioning of the cellular membrane, and therefore affect its physicochemical properties, such as the surface charge. Since this parameter depends on the composition of membrane, any changes in the quantity and quality of charged functional groups on its surface result in changes in surface charge density values, thus affecting membrane-surrounding equilibria. Therefore, observed alterations in values of the determined parameters are likely the result of a number of different phenomena that occur in the cell membrane due to the interactions with p-CoA. The results of our research may be useful in predicting overall influence of p-CoA on membranes of malignant cells, and hopefully in the future will become a useful tool in determination of drug–membrane interactions while assessing therapeutic potential of particular agents. However, further extensive multidirectional analyses are required to reveal detailed systematic effects of p-CoA activity on membranes of living cells to successfully use electrochemical parameters as indicators of pharmacological potential of the drug.

## Figures and Tables

**Table 1:** Effect of p-coumaric acid on the acidic and basic functional groups concentrations and associations constants with H^+^ and OH^-^ ions of glioblastoma cell lines.

Cell Line	Groups	Parameters			
		*C*_TA_ (10^−6^ mol/m^2^)	*C*_TB_ (10^−6^ mol/m^2^)	*K*_AH_ (10 m^3^/mol)	*K*_BOH_ (10^7^ m^3^/mol)
**LN18**	Control (24 h)	4.82 ± 0.09	1.33 ± 0.02	63.30 ± 0.11	1.52 ± 0.09
	+ 5 mM CA (24 h)	5.24 ± 0.10	1.38 ± 0.02	56.10 ± 0.12	1.97 ± 0.08
	+ 8 mM CA (24 h)	5.78 ± 0.09	2.17 ± 0.05	53.20 ± 0.09	3.39 ± 0.11
	Control (48 h)	4.68 ± 0.07	1.54 ± 0.03	134.00 ± 0.11	3.55 ± 0.07
	+ 5 mM CA (48 h)	5.24 ± 0.06	1.75 ± 0.02	123.00 ± 0.07	3.67 ± 0.09
	+ 8 mM CA (48 h)	6.23 ± 0.07	2.03 ± 0.05	81.10 ± 0.10	3.96 ± 0.11
**LN229**	Control (24 h)	3.96 ± 0.08	1.21 ± 0.04	133.00 ± 0.11	2.53 ± 0.02
	+ 5 mM CA (24 h)	5.08 ± 0.07	1.39 ± 0.07	121.00 ± 0.12	2.56 ± 0.02
	+ 8 mM CA (24 h)	6.14 ± 0.09	1.42 ± 0.03	78.40 ± 0.09	3.56 ± 0.05
	Control (48 h)	4.52 ± 0.10	1.02 ± 0.04	121.00 ± 0.08	1.51 ± 0.06
	+ 5 mM CA (48 h)	5.10 ± 0.06	1.11 ± 0.03	112.00 ± 0.06	1.62 ± 0.07
	+ 8 mM CA (48 h)	6.14 ± 0.07	1.41 ± 0.03	98.50 ± 0.07	2.12 ± 0.04
**LBC3**	Control (24 h)	4.88 ± 0.09	1.15 ± 0.05	96.40 ± 0.09	2.38 ± 0.02
	+ 5 mM CA (24 h)	5.31 ± 0.08	1.31 ± 0.06	90.30 ± 0.07	3.51 ± 0.03
	+ 8 mM CA (24 h)	6.00 ± 0.09	1.77 ± 0.09	88.90 ± 0.06	3.83 ± 0.07
	Control (48 h)	4.88 ± 0.06	1.20 ± 0.02	328.00 ± 0.09	1.00 ± 0.08
	+ 5 mM CA (48 h)	5.33 ± 0.04	1.24 ± 0.02	196.00 ± 0.08	1.48 ± 0.09
	+ 8 mM CA (48 h)	5.83 ± 0.10	1.44 ± 0.03	175.00 ± 0.08	2.06 ± 0.06
**T98G**	Control (24 h)	5.75 ± 0.06	1.75 ± 0.03	52.90 ± 0.15	0.70 ± 0.05
	+ 5 mM CA (24 h)	6.29 ± 0.08	1.89 ± 0.05	47.60 ± 0.11	0.97 ± 0.08
	+ 8 mM CA (24 h)	6.68 ± 0.09	2.25 ± 0.08	35.10 ± 0.09	1.87 ± 0.11
	Control (48 h)	5.75 ± 0.06	1.75 ± 0.03	52.90 ± 0.15	0.70 ± 0.05
	+ 5 mM CA (48 h)	6.71 ± 0.09	1.85 ± 0.02	24.90 ± 0.12	0.85 ± 0.06
	+ 8 mM CA (48 h)	6.95 ± 0.10	1.92 ± 0.02	12.80 ± 0.11	1.54 ± 0.07
